# A novel endoscopic ultrasound-guided transluminal anchor device

**DOI:** 10.1055/a-2088-8753

**Published:** 2023-06-12

**Authors:** Abhishek Agnihotri, Alexander Schlachterman

**Affiliations:** 1 Division of Gastroenterology, Bayhealth Medical Center, Dover, Delaware, USA; 2 Division of Gastroenterology, Thomas Jefferson University Hospital, Philadelphia, Pennsylvania, USA

Over the last decade, there has been significant advancement in the field of therapeutic endoscopic ultrasound (EUS) 1 . Multiple transluminal interventions are now performed, including the creation of gastrojejunostomies using a lumen-apposing metal stent (LAMS) and EUS-guided transgastric endoscopic retrograde cholangiopancreatography, among other examples 2 . These procedures carry risks of perforation, which occurs mostly because of stent misdeployment or migration 3 . Attempts to use a 19-gauge needle to place an anchor have been described previously 4 5 ; however, these attempts required manual loading of the anchor, making the procedure cumbersome. We report the use of a novel EUS-guided transluminal anchor and cinch (S-Lock).

An ex vivo porcine stomach model was used to simulate two adjacent lumens ( Video 1 ). A 19-gauge EUS access needle was used to perform transluminal puncture ( Fig. 1 ) – a blunt access needle is preferred to reduce the risk of suture breakage from a sharp needle – and the stylet was removed. The transluminal anchor device was loaded and the steel insert was advanced. Under EUS guidance, the anchor was advanced through the needle and visualized to be in the lumen on EUS. The insert and the 19-gauge needle were then removed, leaving the suture and anchor in place. The suture was then cinched, leading to apposition of the two lumens ( Fig. 2 ). More than one anchor may be applied in this way.

**Video 1** Demonstration in an ex vivo porcine stomach model and step-by-step guide to the use of the device, deploying a transluminal anchor and cinching the suture in place.


**Fig. 1:**
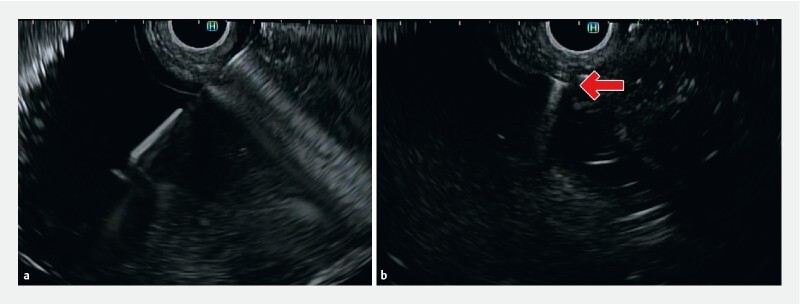
Endoscopic ultrasound images showing: **a** transluminal puncture using a 19-gauge needle; **b** deployment of the anchor in the lumen, with the anchor visualized as a hyperechoic shadowing reflector (red arrow), with tenting of the lumen wall on pulling the attached suture.

**Fig. 2:**
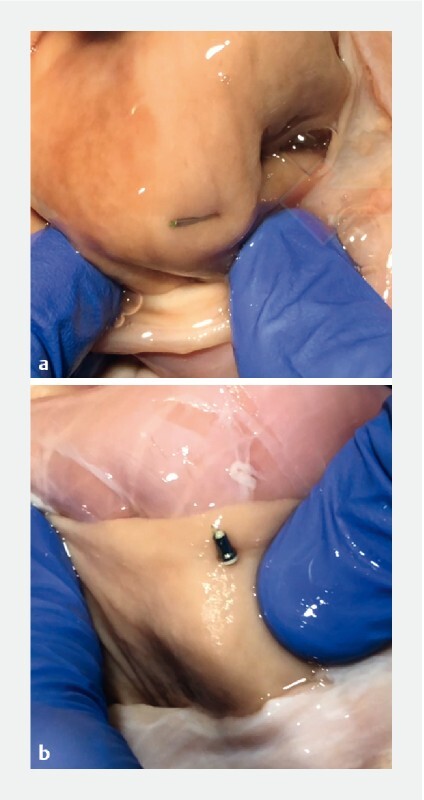
Photographs showing the appearances of: **a** the anchor in the lumen; **b** the cinch in the opposite lumen.

This device significantly improves upon previously described devices, given its single platform with a loaded anchor, which obviates the need to manually load an anchor and suture. The device has the potential to make transluminal interventions using LAMSs safer and reduce the risk of stent misdeployment and migration. Further research is needed on the safety and efficacy of this device in human trials.

Endoscopy_UCTN_Code_TTT_1AS_2AB

## Notes

Endoscopy E-Videos

https://eref.thieme.de/e-videos
*E-Videos* is an open access online section of the journal *Endoscopy* , reporting on interesting cases and new techniques in gastroenterological endoscopy. All papers include a high-quality video and are published with a Creative Commons CC-BY license. Endoscopy E-Videos qualify for HINARI discounts and waivers and eligibility is automatically checked during the submission process. We grant 100% waivers to articles whose corresponding authors are based in Group A countries and 50% waivers to those who are based in Group B countries as classified by Research4Life (see: https://www.research4life.org/access/eligibility/ ). This section has its own submission website at https://mc.manuscriptcentral.com/e-videos
